# H_2_O_2_ and NO_2_^−^ in Exhaled Breath Condensate Increase After a Wheelchair Rugby Match in Paralympic Athletes: A Possible Effect of Functional Classification

**DOI:** 10.3390/antiox15060705

**Published:** 2026-06-03

**Authors:** Cristián Rosales-Antequera, Sebastián Caballero, Ginés Viscor, Teresa Carbonell, Oscar F. Araneda

**Affiliations:** 1 Integrative Laboratory of Biomechanics and Physiology of Effort (LIBFE), Kinesiology School, Faculty of Medicine, Universidad de los Andes, Monseñor Álvaro del Portillo 12455, Las Condes, Santiago 7550000, Chile; carosales@miuandes.cl; 2 Physical Medicine and Rehabilitation Unit, Clínica Universidad de los Andes, Santiago 8320000, Chile; 3 Physiology Section, Department of Cell Biology, Physiology and Immunology, Faculty of Biology, Universitat de Barcelona, 08028 Barcelona, Spain; gviscor@ub.edu (G.V.); tcarbonell@ub.edu (T.C.); 4 Kinesiology and Occupational Therapy Unit, Hospital Padre Hurtado, Santiago 8880465, Chile; sebastiancaballero.c@gmail.com; 5 Wheelchair Rugby National Team, Chile Paralympic Committee, Ñuñoa 7750000, Chile

**Keywords:** spinal cord injury, wheelchair rugby, exhaled breath condensate, hydrogen peroxide, nitrite

## Abstract

Spinal cord injury (SCI) is associated with respiratory dysfunction, chronic inflammation, and oxidative stress, and can increase pulmonary tissue stress during exercise. Thus, hydrogen peroxide (H_2_O_2_) and nitrite (NO_2_^−^) concentrations in exhaled breath condensate (EBC) were compared during an official wheelchair rugby match. 14 males and two females with SCI (33.3 ± 6.5 years), anthropometry, baseline spirometry, and the International Wheelchair Rugby Federation classification (IWRF) were recorded. Playing time (23.6 ± 7.49 min), Borg scale (4.3 ± 1.64), and [H_2_O_2_] EBC and [NO_2_^−^] EBC were determined before and 20 min after the match. In the total sample, [H_2_O_2_] EBC and [NO_2_^−^] EBC increased post-match (*p* = 0.0042 and *p* = 0.031, respectively). When segmented according to IWRF classification, the highest functional capacity, H group (>1.5 points; n = 11) increased its [H_2_O_2_] EBC per exercise (*p* = 0.0029) and showed a trend for [NO_2_^−^] EBC (*p* = 0.09), while the lowest classification, L group (≤1.5 points; n = 5) showed a higher baseline concentration in both EBC markers with no changes per exercise. Baseline IWRF classification was inversely correlated with [H_2_O_2_] EBC, while body mass index (BMI) was positively associated with [NO_2_^−^] EBC. In conclusion, in the sample analyzed, a short period of moderate intensity during a wheelchair rugby match increases H_2_O_2_ and NO_2_^−^ in the airway with a potentially greater effect in SCI athletes with better functional capacity; it remains to be determined whether this phenomenon corresponds to a physiological or pathological process.

## 1. Introduction

Spinal cord injury (SCI) causes muscle paralysis and loss of sensation as a result of damage to the neurons that regulate these functions [1,2]. However, its effects go beyond a simple locomotor disorder, as this condition involves multisystemic alterations [3,4]. Among the main consequences are compromised neurovegetative control, increased susceptibility to infections, impaired thermal control, chronic inflammation, oxidative stress, and compromised lung function, among others [5,6,7,8,9].

The consequences of SCI on the respiratory system are diverse and vary in severity, depending mainly on the level of injury, which determines the degree of involvement of the respiratory muscles and the protective mechanisms of the respiratory system [10,11]. On the other hand, the level of injury determines the compromise of autonomic control, leading to greater stress on the vascular walls, which predisposes to atherosclerosis and uncontrolled catecholamine release, thereby maintaining a chronic proinflammatory state [9,12]. Thus, several studies have reported that individuals with SCI have restrictive spirometric patterns due to respiratory muscle weakness, increased chest stiffness, and poor abdominal control. In addition to this, some studies mention that a portion of subjects with SCI have an obstructive spirometric pattern, similar to that of asthmatic patients [13]. The possible causes of this obstructive pattern are attributed to denervation of the sympathetic component of the airways, which generates an autonomic imbalance, and to an increase in adipokines that can induce a proinflammatory effect in the respiratory system, exacerbated by chronic damage to the lung parenchyma due to decreased lung compliance [10,14,15,16,17,18]. It has been observed that this obstructive pattern in subjects with SCI may be accompanied by elevated plasma levels of inflammatory mediators, such as C-reactive protein and IL-6, suggesting that an inflammatory reaction may underlie this pattern [19]. This compromise of the respiratory system is accompanied by local alterations, including decreased mucociliary clearance, changes in the microbiota, increased incidence of upper respiratory tract infections, and microaspirations that stimulate a local inflammatory environment [20,21]. In this context, to assess whether there is indeed a local increase in inflammatory markers in the respiratory system, Radulovic et al., using a non-invasive technique to measure exhaled nitric oxide, observed that subjects with quadriplegia showed higher levels of exhaled nitric oxide than a healthy control group, similar to those of asthmatic subjects [22].

West et al., meanwhile, observed in the sporting context that athletes with SCI at rest maintain a restrictive pattern compared to healthy athletes [9,23]. The fact that people with SCI who participate in physical training programs maintain restrictive breathing patterns reinforces the hypothesis that, during physical activity, they may be exposed to greater pulmonary tissue stress than healthy subjects due to exercise-induced hyperventilation [24]. This makes it relevant to evaluate the response of inflammatory and oxidative stress markers in the lungs of individuals with SCI who exercise. To examine the local effect of exercise on the respiratory system in athletes with spinal cord injury, analyses can be conducted on condensed exhaled breath (EBC) samples to assess levels of reactive chemical species markers of the local redox state, as modulated by physical exertion, along with associated inflammatory mediators. This methodology has the advantage of being non-invasive, allowing the analysis of these markers of inflammation and oxidative stress from a sample obtained directly from the respiratory system, a method widely used by our research group in conventional athletes [25,26,27,28].

Thus, as previously stated, the objective of this study was to analyze the behavior of H_2_O_2_ and NO_2_ concentrations in EBC samples from wheelchair rugby players under real game conditions and, secondarily, to determine the influence of the IWRF (International Wheelchair Rugby Federation) Classification on these markers.

## 2. Materials and Methods

### 2.1. Study Population

Data were collected during official wheelchair rugby matches involving 16 players, 14 men and two women (see Table 1 for general characteristics). Among the participants, eight competed at the South American level (national team members), while the remaining eight were local-level players in the discipline. Their training load was 7.34 ± 2.89 times per week (see Supplementary Table S1). In wheelchair rugby, athletes are grouped to compete according to the IWRF Classification by the federation expert certifiers. The parameters evaluated include muscle strength, range of motion, grip, grasp, and wheelchair push. This scale is used to obtain a score between 0.5 and 3.5 [29]. The values obtained were used both as a whole for correlations with other parameters and through their a priori segmentation. Thus, a first group (group L: Low classification, five males) was formed using a classification value of <1.5 as the criterion, while group H (High classification, two females and nine males) had classification values >1.5.

Twenty-five percent of participants were smokers (see Supplementary Table S2). Regarding spirometric analysis, 37.5% showed a restrictive pattern and 12.5% an obstructive pattern. The individual values can be found in the Supplementary Table S3. From a nutritional standpoint, 50% were within a normal weight range, 43.7% were overweight, and 6.25% were obese. Regarding medical history, 25% reported having had a urinary tract infection, 25% had lung problems (pneumonia, pleurodesis, and asthma), and 12.5% had other problems (cholelithiasis, bone fistula). Details of these disorders are provided in the Supplementary Table S2.

Before obtaining the samples, the athletes were informed of the study’s objectives and procedures through an oral presentation. They then completed and signed the individual informed consent form. The inclusion criteria were being a wheelchair rugby player, with a spinal cord injury of more than six months’ duration, with a minimum training frequency of once a week, and at least six months’ experience in the sport. Exclusion criteria were the consumption of antioxidants, the presence of both lower (more than six months) and upper (more than three weeks) respiratory pathologies, and a current urinary tract infection. The research protocol adhered to the ethical principles outlined in the Declaration of Helsinki for research involving human subjects. It was approved by the Scientific Ethics Committee of the Universidad de los Andes, Chile (Project No. CEC2024025).

### 2.2. Protocol

For this experiment, the participants’ anthropometric measurements were first recorded. They also underwent baseline spirometry to assess their lung function before the match. In addition, since it is known that cigarettes are an inflammatory agent in the airways, participants were asked to refrain from smoking 24 h before the evaluation. The EBC was collected using a device previously used by our research group in both healthy individuals and conventional athletes [30,31]. Thus, before the match began, the participants were placed in a room adjacent to the sports venue, where they sat comfortably in their respective wheelchairs and remained at rest with a nasal clip. The players breathed through the device described in a constant, rhythmic manner, without forcibly altering their respiratory rate. The procedure lasted 15–20 min and collected approximately 1.5 mL of EBC. The second sample was collected from all subjects at least 20 min after the end of the match, based on the fact that we had previously observed differences at this time point in participants in running races and in laboratory cycling exercises [25].

**Figure 1:**
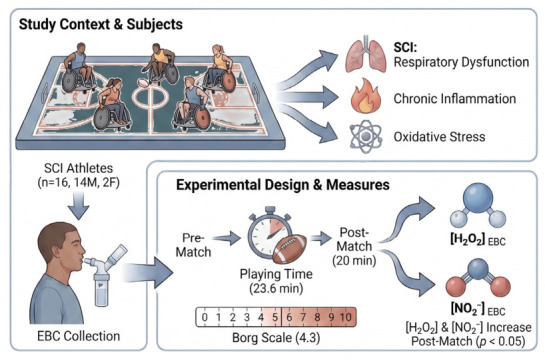
Sixteen wheelchair rugby players (14M/2F) during official rugby matches. Baseline spirometry values, playing time, perceived exertion (Borg scale), and H_2_O_2_ and NO_2_^−^ concentrations in EBC samples collected before and 20 min after the match were determined.

A summary of the experimental protocol is presented in Figure 1.

*[NO_2_ˉ] in EBC:* To determine the nitrite concentration in EBC, a reaction using Griess reagent (Sigma-Aldrich, St. Louis, MO, USA) was employed [32]. To do this, 300 μL of Griess reagent, prepared with 0.1% N-(1-naphthyl)ethylenediamine dihydrochloride, 1% sulfanilamide, and 3% H_3_PO_4_, was added to 300 μL of EBC. Once the reaction had occurred, the absorbance was measured at 550 nm after 10 min of incubation at room temperature. To obtain the calibration curve, the same procedure was followed as for the EBC samples, with three tests conducted at concentrations of sodium nitrite (Sigma-Aldrich, St. Louis, MO, USA) ranging from 0.5 to 10 μM.

*[H_2_O_2_] EBC:* Its determination was based on the reaction of the FOX2 reagent in an acidic medium [33]. The FOX2 reagent consists of Fe^2+^ (250 μM) diluted in 110 mM HClO_4_ in the presence of 100 mM sorbitol according to Gay and Gebicki [34]. Finally, the reaction of FOX2 with H_2_O_2_ (sample/standard) induces the oxidation of the metal in the reagent, which is monitored with the xylenol orange indicator (250 μM) that changes from orange to purple. For the determination and construction of the calibration curve, 150 μL of FOX2 and 350 μL of EBC/standard were used, and the absorbance of the mixture was read at 560 nm (Jenway 6405, Jenway Ltd., Dunmow, Essex, UK) after 1 h of incubation at room temperature in the dark. To construct the calibration curve, three tests were performed at H_2_O_2_ (Merck KGaA, Darmstadt, Germany) concentrations from 0.05 to 10 μM.

During the match, the modified Borg scale, which ranges from 0 to 10, was used to assess perceived exertion [35]. Each player was asked to rate their perception according to this score at the end of each period of play (eight minutes) or when the player was replaced during the game. In addition, the number of minutes played on the court was recorded using an individual timing system. Finally, by multiplying both parameters (Borg × time), the individual total workload during the match was determined [36].

### 2.3. Statistical Analysis

The Shapiro–Wilk test was used to study the distribution of the variables analyzed. Parameters that were normally distributed are expressed as mean ± standard deviation, while those that were distributed differently are represented as median and interquartile range in parentheses. The comparison of H_2_O_2_ and NO_2_^−^ concentrations between pre- and post-match values for both the total group and the sample segmented according to the IWRF Classification was performed using the Wilcoxon test; meanwhile, the Mann–Whitney test was used to compare the baseline values of the two EBC biomarkers. Finally, Spearman’s rank correlation coefficient (ρ) was calculated to evaluate potential correlations between variables. All the tests were performed at α = 0.05 in Excel (Microsoft Corporation, Redmond, WA, USA) and GraphPad Prism 10 (GraphPad Software LLC, Boston, MA, USA).

### 2.4. Sample Size Estimation and Justification

The required sample size was estimated a priori using data reported by Araneda O.F. et al. [25], who assessed changes in exhaled breath condensate before and after physical exercise in healthy athletes, as there are no similar studies in Paralympic athletes. The calculation was performed using EBC [H_2_O_2_] values. Sample size estimation was conducted using G*Power software (Heinrich Heine University Düsseldorf, Germany; version 3.1.9.7). Based on the expected pre–post differences, a Wilcoxon signed-rank test for paired samples was selected. The parameters used for the calculation included an effect size of 1.28, an alpha error probability of 0.05, and a statistical power of 0.80, resulting in a required minimum sample of eight participants. Considering feasibility and participant availability during the competitive period, the final sample size was increased, and sixteen participants were ultimately included in the study, thereby exceeding the minimum number required to detect the expected effect. Furthermore, it should be noted that the study was conducted in a highly specific adapted sport population, which inherently limits the pool of eligible participants; however, the repeated-measures design and the final sample size exceeding the a priori requirement strengthen the statistical sensitivity to detect meaningful exercise-induced changes.

## 3. Results

The average IWRF Classification was 2.03 ± 1.04 pts (see individual descriptions in Supplementary Table S1). The average obtained for group L was 0.8 ± 0.44 pts, while for group H it was 2.59 ± 0.66 pts (*p* = 0.0002). During the matches, the players remained in effective play time for 23.6 ± 7.49 min and displayed a Borg score of 4.3 ± 1.64; thus, the physical load, expressed as Borg × time, was 101.34 ± 12.34.

**Figure 2:**
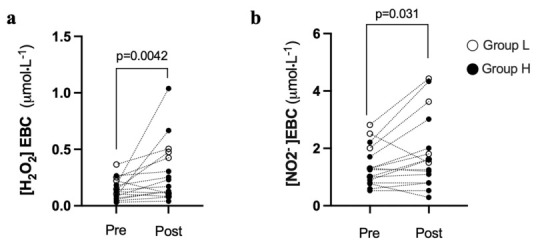
[H_2_O_2_] EBC (**a**) and [NO_2_^−^] EBC (**b**) before and after a wheelchair rugby match (n = 16). Each point represents one participant, and the dotted lines connect paired values. Group L has an IWRF Classification ≤ 1.5 points, and group H has a rating >1.5 points. Medians were compared using the Wilcoxon test.

The parameters measured in the EBC are shown in Figure 2, which presents the individual values before and after the match for [H_2_O_2_] EBC (Figure 2a) and [NO_2_^−^] EBC (Figure 2b), respectively. In the case of [H_2_O_2_] EBC, we observed an increase after exercise, with mean values of 0.13 (0.07–0.21) µM/L and 0.20 (0.11–0.46) µM/L before and after the match, respectively. For nitrite, the same pattern was observed, with pre-exercise values of 1.28 (0.82–1.93) µM/L and post-exercise values of 1.55 (0.87–2.77) µM/L.

Segmented analysis of the sample showed that group L had [H_2_O_2_] EBC pre-exercise values of 0.22 (0.13–0.30) µM/L and post-exercise values of 0.43 (0.10–0.49) µM/L with no evidence of statistically significant differences (*p* = 0.31). Similarly, no changes (*p* = 0.31) due to exercise were observed in [NO_2_^−^] EBC, with pre-exercise values of 2.01 (1.16–2.67) µM/L and post-exercise values of 1.80 (1.56–4.03) µM/L. In turn, in group H, there was a significant exercise-induced increase in [H_2_O_2_] EBC (*p* = 0.0029), with pre-exercise values of 0.087 (0.06–0.18) µM/L and post-exercise values of 0.17 (0.11–0.30) µM/L. The same trend (*p* = 0.09) was found in this group for [NO_2_^−^] EBC, with pre-exercise values of 1.00 (0.77–1.30) µM/L and post-exercise values of 1.20 (0.80–2.01) µM/L. The medians for baseline values were also higher in the L group than in the H group for both EBC biomarkers, with *p* = 0.048 for [H_2_O_2_] EBC and *p* = 0.037 for [NO_2_^−^] EBC. Within the segmented analysis, it was also found that the average playing time for the L group was 18.80 ± 5.7 min, while in the H group the average playing time was 25.64 ± 7.41 min, with a tendency toward difference between the two groups (*p* = 0.09). However, the Borg mean in group L was 4.8 ± 1.9, while in group H it was 4.09 ± 1.6, with a *p* = 0.45. The physical load to which both groups (calculated as the Borg index × time) were exposed resulted in an average of 96.2 ± 68.57 for group L and 106 ± 51.43 for group H (*p* = 0.75).

**Figure 3:**
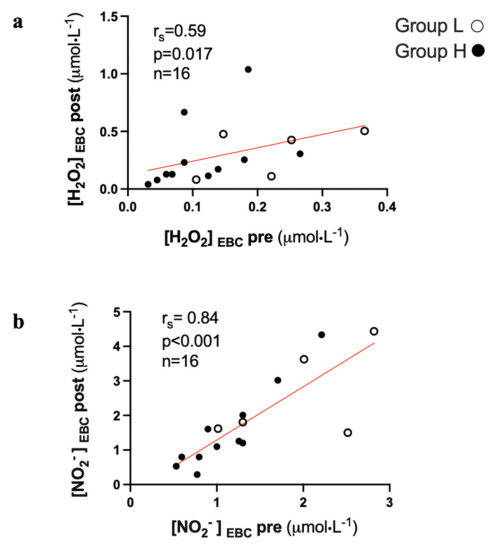
Correlations between pre- and post-match values of [H_2_O_2_] EBC (**a**) and [NO_2_^−^] EBC (**b**) in a wheelchair rugby match. Group L has an IWRF classification of ≤1.5 points, and group H has a classification of >1.5 points. Spearman’s rho (ρ) was determined for the correlations.

**Figure 4:**
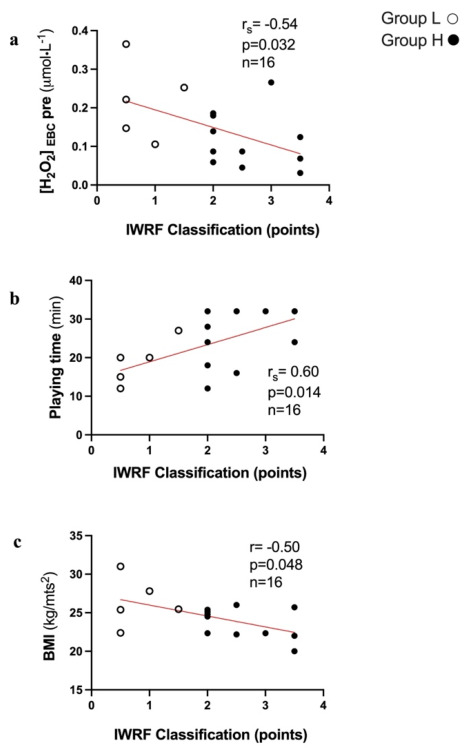
Correlation between the IWRF Classification and pre-exercise [H_2_O_2_] EBC (**a**), real playing time (**b**), and body mass index (**c**). The L group has an IWRF Classification of ≤1.5 points, and the H group has a classification of >1.5 points. Spearman’s rho (ρ) was used to determine the correlations.

Regarding correlations, a significant association was found in the total group between pre- and post-match values of [H_2_O_2_] EBC and [NO_2_^−^] EBC (see Figure 3a and Figure 3b, respectively). In addition, a significant association was found between the absolute changes in both variables (ρ = 0.58, *p* = 0.018, n = 16). In another aspect, the IWRF Classification of the athletes was correlated with both parameters, indicating inflammation and oxidative damage, as well as the indicators obtained during the match. Thus, we found an inverse correlation between the IWRF Classification and the pre-match baseline [H_2_O_2_] EBC values (Figure 4a). In turn, a trend was observed between pre-game [NO_2_^−^] EBC and IWRF Classification values (ρ = −0.43, *p* = 0.09). On the other hand, the IWRF Classification showed a direct relationship with playing time and an inverse relationship with BMI (see Figure 4b and Figure 4c, respectively). In addition, the BMI variable correlated inversely with the FEV1/FVC ratio (ρ = −0.54, *p* = 0.032) and directly with basal nitrite (ρ = 0.53, *p* = 0.036).

## 4. Discussion

The spinal cord injury not only compromises respiratory motor function but also induces a chronic inflammatory state that directly affects the lungs. Thus, alterations in autonomic regulation and respiratory mechanics promote the sustained release of low levels of proinflammatory cytokines and increased oxidative stress in the airways. Even in low chest injuries, increased pulmonary permeability, tissue infiltration of neutrophils/macrophages, and an exacerbated response to proinflammatory stimuli have been observed, which can accelerate functional deterioration of the respiratory system [12,37]. The present study shows that a wheelchair rugby match increases [H_2_O_2_] and [NO_2_^−^] in post-exercise EBC in the overall group of participants. Additionally, it is possible that the magnitude of these changes is greater in players with a higher IWRF Classification specific to this sport and low spinal cord injury. This finding confirms that competitive exercise under real game conditions increases H_2_O_2_ and NO_2_^−^ in the airways in this population, a phenomenon previously described in conventional athletes [25,28,38] but not documented until now in athletes with SCI.

Hydrogen peroxide is an important marker of inflammation because of its ability to participate in reactions that induce oxidation of biomolecules. This compound is formed by the reaction of superoxide radicals (O_2_^−^) with protons (H^+^), a reaction catalyzed by the enzyme superoxide dismutase. Several cells can produce it. Specifically, in the respiratory system, phagocytic cells, type II pneumocytes, and airway epithelial cells have this ability [39,40,41]. Our research group has contributed by measuring this reactive species as a marker during physical exercise [25,28]. However, measuring this in the exhaled air of athletes with spinal cord injuries after performing their sport is an innovative assessment. This approach provides new information for a deeper understanding of exercise physiology in Paralympic athletes. On the other hand, nitrite (NO_2_^−^) is considered another oxidizing agent produced by the metabolism of nitric oxide (NO). It has the advantage of greater stability, which allows it to be detected in exhaled air [42]. When NO reacts with reactive oxygen species, it can generate NO_2_^−^, a highly oxidizing molecule. For example, in asthma, cytokines produced by Th2 lymphocytes (such as IL-4 and IL-13) induce NO production in bronchial epithelial cells, leading to higher NO_2_^−^ concentrations [43,44]. Although NO has a bronchodilator effect, in the context of asthma, excessive production is associated with oxidative damage, epithelial dysfunction, and persistent inflammation [45,46]. Notwithstanding the above, we must not forget that the metabolism of NO derivatives is complex; they do not exclusively promote oxidative damage, since NO_2_^−^ is part of a metabolic pathway that is difficult to interpret. For this reason, we cannot rule out the possibility that an increase in NO_2_^−^ might be due to a physiological phenomenon, given that nitric oxide is known to increase after exercise.

To our knowledge, this report is the only one to date that has studied the behavior of this marker in EBC under physical exertion in athletes with spinal cord injury. Analysis of our results shows an increase in [H_2_O_2_] EBC and [NO_2_^−^] EBC after the game. The changes observed suggest that exercise, under these conditions, activates local oxidative mechanisms, probably associated with increased ventilatory activity, mechanical stress, epithelial desiccation, and a drop in airway temperature, which a systemic oxidative imbalance may partly explain. This phenomenon has been described by our research group in disciplines with high ventilatory demands, such as swimming or long-distance running [25,28]. However, the present study provides evidence that it also occurs in high-intensity adapted sports, albeit at lower ventilatory loads and demands in athletes who are known to have weaker respiratory muscles and, as a result, a lower tidal volume and maximum respiratory rate, particularly those with higher-level spinal cord injuries [47]. Another important point is that our sample included four tobacco smokers, a substance known to have a pro-inflammatory effect on the airways. Three participants smoked fewer than five cigarettes per day, and one smoked fifteen per day; they were randomly distributed across the two groups in the segmented analysis, with one participant having a normal spirometric pattern and another having a restrictive pattern in each group (see Tables S2 and S3). Although we cannot rule out the possibility that this substance may have interfered with the results, participants were asked to stop smoking beforehand (see Section 2), and their results were evaluated individually a posteriori using the criterion that baseline values of [H_2_O_2_] EBC and [NO_2_^−^] EBC should not deviate significantly from the median.

One characteristic of the population under study is its interindividual diversity. For this reason, we performed a segmented analysis of the sample, using the IWRF Classification criteria. Thus, [NO_2_^−^] in EBC in group H (lower lesion height) showed a tendency to increase after exercise without reaching significance (*p* = 0.09), while in group L (higher lesion height), no differences were observed. About [H_2_O_2_] EBC, it was observed that group H increased [H_2_O_2_] EBC due to exercise, while no differences were found in group L (see Section 3). Regarding these findings, we expected to find the opposite, since higher injuries are associated with greater autonomic and ventilatory compromise and, therefore, greater chronic epithelial inflammatory susceptibility [5,6,48]. Different factors could explain this phenomenon. First, group L had higher baseline values for both markers, which could have reduced the acute response to exercise if the athletes were already in a baseline state of inflammation at the start of the test. In line with this, Figure 3a shows that pre-exercise [H_2_O_2_] EBC levels determine the values reached post-exercise. Furthermore, when correlating pre-exercise values with absolute changes, we found a trend only for [NO_2_^−^] EBC (r = 0.44, *p* = 0.08). In addition to the above, there is a negative correlation between the IWRF Classification and baseline levels of [H_2_O_2_] EBC (see Figure 4a), which may be associated with the fact that players with lower functional capacity may have greater chronic oxidative stress, as has been previously described in people with high spinal cord injuries [49,50]. Finally, another factor that could influence this phenomenon is exercise-related factors such as load and total work time, which can also determine the magnitude of the oxidative response to exercise [51]. In this regard, we observed that the exercise time in group H was 36% higher than in group L, but did not reach statistical significance (*p* = 0.09). An alternative analysis to the functional classification was performed by segmenting groups based on their spirometric pattern (normal versus pathological), with eight participants in each group (see Table S3). Thus, we found differences only in the baseline [NO_2_^−^] EBC values (*p* = 0.049), with no significant association with other variables. In this regard, we believe this reinforces the idea that grouping participants based on the IWRF provides a more comprehensive view of their functional status, which is why it was ultimately chosen for our analysis.

On the other hand, a higher body mass index (BMI) was associated with a lower FEV_1_/FVC ratio, which has been linked to a tendency toward bronchial obstruction. This association suggests that excess adipose tissue could promote a pro-inflammatory pulmonary environment, in line with the concept of low-grade chronic inflammation induced by adipokines described in the literature [4,9]. Furthermore, evidence of this phenomenon may be the direct correlation observed between BMI and pre-EBC [NO_2_^−^] (see Section 3). Along with obesity, other factors may have influenced our results, such as the presence of respiratory and systemic comorbidities in a part of the sample, which may have acted as a potentially modulating factor of acute changes due to exercise [22]. Although criteria were used to rule out acute pathologies or decompensation of chronic conditions that could influence the measurements, the spinal cord injury patient population and the high-performance athlete sample are heterogeneous. For these reasons, we established for recruitment that the injury had to be older than six months to reduce the inflammatory impact of the traumatic event; for lower respiratory conditions, a recovery period of six months was also an exclusion criterion, whereas three weeks was required for upper respiratory conditions.

This study has limitations that should be considered. The small sample size and population heterogeneity make it hard to generalize the findings, particularly in the case of segmented analysis, due to the low statistical power associated with the small number of participants per group (five versus eleven participants). Secondly, exposure to physical exertion was not homogeneous among participants, as playing time and intensity vary according to tactical position, game plan, athletes’ physical capacity, and coaching decisions. Although the data were obtained under more realistic conditions, this also means that each player was subjected to a variable workload, which may have influenced our results. On another note, while EBC analysis offers a non-invasive assessment method, there are methodological aspects that need improvement to ensure reliable measurements; nevertheless, it has proven useful in serial samples such as those presented in this report. Finally, our study included variables that partially describe changes in redox and inflammatory status, which should subsequently be validated and expanded to include these athletes under laboratory conditions and standardizing timing and physical load.

## 5. Conclusions

A wheelchair rugby match increases [H_2_O_2_] and [NO_2_^−^] levels in EBC with a potentially more pronounced effect in players with low spinal cord injuries, which should be further explored under more controlled conditions. These findings suggest that competitive physical activity in this population can trigger a local inflammatory response in the lungs, in line with what has been described in conventional athletes, and consistent with the greater systemic/pulmonary inflammatory susceptibility described in individuals with spinal cord injury, but at a lower absolute exercise load, although at this point we cannot rule out the possibility that it is a physiological phenomenon. EBC analysis is projected to be a useful, non-invasive, and sensitive tool for assessing the impact of exercise on oxidative stress at the pulmonary level and to design measures to mitigate this lung inflammatory phenomenon.

## Figures and Tables

**Table 1:** General description of participants.

	Values	Predicted Values (%)
Age (yrs)	33.31 ± 6.48	
Height (m)	1.77 ± 0.09	
Body Weight (kg)	77.56 ± 12.21	
BMI (kg/m^2^)	24.52 ± 2.67	
FVC (L)	4.71 ± 1.82	90 ± 46.69
FEV_1_ (L)	3.7 ± 0.94	86 ± 32.05
FEV_1_/FVC (%)	81.72 ± 13.33	115 ± 25.49

Values of the 16 participants are expressed as mean ± SD. BMI *=* body mass index, FVC = forced vital capacity, FEV_1_ = forced Expiratory Volume in one second.

## Data Availability

The original contributions presented in this study are included in the article and Supplementary Materials. Further inquiries can be directed to the corresponding authors.

## Supplementary Materials

The following supporting information can be downloaded at https://www.mdpi.com/article/10.3390/antiox15060705/s1. Table S1: Sports Activity Background of the Participants; Table S2: Spinal Cord Injury Characteristics, Medical History, and Smoking Status. Table S3: Spirometric values.

## Abbreviations

The following abbreviations are used in this manuscript:

BMI	Body Mass Index
EBC	Exhaled Breath Condensate
FEV_1_	Forced Expiratory Volume in One Second
FVC	Forced Vital Capacity
H_2_O_2_	Hydrogen Peroxide
IWRF	International Wheelchair Rugby Federation
NO	Nitric Oxide
NO_2_^−^	Nitrite
ROS	Reactive Oxygen Species
SCI	Spinal Cord Injury
