# Supplementary material for: H2O2 and NO2− in Exhaled Breath Condensate Increase After a Wheelchair Rugby Match in Paralympic Athletes: A Possible Effect of Functional Classification
Source: Antioxidants (Basel). 2026 Jun 3;15(6):705. doi: 10.3390/antiox15060705 (PMC13295657; doi:10.3390/antiox15060705)
Supplement: Supplementary file 1 [file antioxidants-15-00705-s001.zip › antioxidants-4296041-supplementary.pdf]

Supplementary Tables

Article Title: H_2O_2 and NO_2^- in exhaled breath condensate increase after a wheelchair rugby match in Paralympic athletes, depending on their functional classification.

Cristián Rosales-Antequera[1,2,3], Sebastián Caballero[4,5], Ginés Viscor[3], Teresa Carbonell[3] and Oscar F. Araneda[1*].

[1] Integrative Laboratory of Biomechanics and Physiology of Effort (LIBFE), Kinesiology School, Faculty of Medicine, Universidad de los Andes, Monseñor Álvaro del Portillo, Las Condes, Santiago 12455, Chile.

[2] Physical Medicine and Rehabilitation Unit, Clínica Universidad de los Andes, Santiago 8320000, Chile

[3] Physiology Section, Department of Cell Biology, Physiology, and Immunology, Faculty of Biology, Universitat de Barcelona, 08028 Barcelona, Spain

[4] Kinesiology and Occupational therapy unit, Hospital Padre Hurtado, Santiago 8880465, Chile

[5] Wheelchair Rugby National Team, Chile Paralympic Committee, Ñuñoa 7750000, Chile

*Correspondence: ofaraneda@miuandes.cl

Table S1: Sports Activity Background of the Participants.

Participant	IWRF Classification (Points)	Competitive level	Training Frequency (times per week)
1	0.5	South American	3
2	0.5	South American	3
3	0.5	Local	2
4	1	South American	5
5	1.5	South American	2
6	2	South American	3
7	2	Local	3
8	2	South American	3
9	2	South American	5
10	2	Local	5
11	2.5	South American	3
12	2.5	Local	2
13	3	Local	5
14	3.5	South American	1
15	3.5	Local	2
16	3.5	Local	1

IWRF = International Wheelchair Rugby Federation. Participants with numbers from one to five belong to Group L, while those with numbers from 6 to 16 belong to Group H.

Table S2: Spinal Cord Injury Characteristics, Medical History, and Smoking Status.

Participant	Sex	Age	Type of injury	Years since injury	Medical history	Number of cigarettes per day
1	Male	28	C5-C6 (Complete)	8	Recurrent urinary tract infection	1
2	Male	33	C6-C7 (Complete)	2	Recurrent urinary tract infection	0
3	Male	49	C5-C6 (Complete)	8	Recurrent urinary tract infection	0
4	Male	35	SCI C5 (Incomplete)	2	Previously treated bone fistula	5
5	Male	34	C6-C7 (Complete)	9	Recurrent urinary tract infection	0
6	Male	27	C6-C7 (Complete)	9	Pneumonia and Asthma	0
7	Male	29	C6-C7 (Incomplete)	5		0
8	Male	31	C4-C5 (Incomplete)	10	Pneumonia	15
9	Male	30	C6-C7 (Complete)	3		0
10	Male	39	C6-C7 (Incomplete)	2	Pneumonia	0
11	Male	33	C7-C8 (Complete)	30		4
12	Female	33	C5-C6 (Incomplete)	17		0
13	Male	33	C7-T1 (Complete)	4		0
14	Male	24	T1-T2 (Complete)	9	Unilateral pleurodesis	0
15	Male	45	T5-T6 (Complete)	22		0
16	Female	30	T10-T12 (Complete)	8	Cholelithiasis	0

Participants with numbers from one to five belong to Group L, while those with numbers from 6 to 16 belong to Group H.

Table S3: Spirometric values.

Participant	FEV1 (l)	FVC (l)	FEV1/FVC (%)	Spirometry Pattern
1	2.94	2.94	100	Restrictive
2	2.87	3.31	86.7	Restrictive
3	5.03	7.05	71.3	Normal
4	3.73	3.81	97.9	Restrictive
5	3.62	5.1	71	Normal
6	3.14	5.41	59	Obstructive
7	4.35	5.03	86.4	Normal
8	3.56	4.75	74.9	Normal
9	4.31	5.36	80	Normal
10	3.24	4.19	77	Normal
11	2.82	3.1	90.9	Restrictive
12	6.37	10.8	58.9	Obstructive
13	3.88	4.15	93	Restrictive
14	2.35	3.02	77.8	Restrictive
15	3.84	3.86	99.4	Normal
16	4.26	4.35	97.9	Normal

Participants with numbers from one to five belong to Group L, while those with numbers from 6 to 16 belong to Group H.
